# Advanced Catalytic Peroxymonosulfate Activation via Zeolite-Supported Cu_3_Mn-Layered Double Hydroxide for Enhanced Oxidative Degradation of Bisphenol A (BPA)

**DOI:** 10.3390/toxics13100889

**Published:** 2025-10-17

**Authors:** Qiuyi Li, Chongmin Liu, Meina Liang, Mi Feng, Zejing Xu, Dunqiu Wang, Saeed Rad

**Affiliations:** 1 College of Environmental Science and Engineering, Guilin University of Technology, Guilin 541004, China; lqy1626743718@163.com (Q.L.);; 2 Guangxi Key Laboratory of Environmental Pollution Control Theory and Technology, Guilin University of Technology, Guilin 541006, China; 3 University Engineering Research Center of Watershed Protection and Green Development, Guangxi, Guilin University of Technology, Guilin 541006, China; 4 Key Laboratory of Carbon Emission and Pollutant Collaborative Control, Education Department of Guangxi Zhuang Autonomous Region, Guilin University of Technology, Guilin 541006, China

**Keywords:** layered double hydroxides, peroxymonosulfate activation, zeolite, zeolite/Cu_3_Mn-LDH, bisphenol A

## Abstract

The widespread presence of bisphenol A (BPA), a persistent endocrine-disrupting pollutant, in aquatic environments poses significant ecological and health risks, necessitating its effective removal. However, conventional remediation technologies are often hampered by catalysts with narrow pH adaptability and poor stability. In this study, a novel catalyst, Zeolite-supported Cu_3_Mn-layered double hydroxide (LDH), was fabricated using the co-precipitation method. The synthesized catalyst was applied to activate peroxymonosulfate (PMS), effectively enabling decomposition of BPA by advanced oxidation processes. The composite material was characterized by X-ray diffraction (XRD), Fourier-transform infrared spectroscopy (FTIR), and transmission electron microscopy (TEM), which confirmed the successful synthesis of the zeolite-supported Cu_3_Mn-LDH. The catalyst exhibited high activity in both neutral and strongly alkaline environments, achieving complete degradation of 10 mg⋅L^−1^ bisphenol A (BPA) within 40 min and a 98% total organic carbon (TOC) removal rate when both the PMS and catalyst were dosed at 0.15 g⋅L^−1^. Singlet oxygen was detected as the primary reactive species responsible for BPA degradation, as verified by quenching experiments and EPR analysis, which also identified the presence of sulfate (SO_4_^•−^), hydroxyl (•OH), and superoxide (•O_2_^−^) radicals. The catalyst exhibited excellent reusability, maintaining high catalytic efficiency over two consecutive cycles with minimal performance loss. Gas chromatography-mass spectrometry (GC-MS) analysis revealed five intermediate products, enabling the proposal of potential BPA degradation pathways. This work not only presents a novel synthetic approach for zeolite-supported LDH composites, but also offers a promising strategy for the efficient removal of BPA from aqueous systems through AOPs.

## 1. Introduction

Bisphenol A (BPA), a common industrial compound used in synthesizing polycarbonate plastics and epoxy resins, poses significant environmental and health risks, owing to its endocrine-disrupting properties [1]. BPA can mimic or antagonize natural hormones, leading to endocrine system dysfunction and a range of adverse effects, including reproductive toxicity, metabolic disorders, and neurodevelopmental abnormalities [2]. Due to the widespread production and use of BPA, environmental contamination has increased, making its removal from aquatic environments an urgent concern. Studies have reported BPA concentrations in anthropogenic wastewater ranging from 16 to 1465 ng·L^−1^, and concentrations varying from 170 to 3113 ng·L^−1^ in surface water [3]. In specific water bodies, BPA has been identified as a major pollutant; for example, in Lake Taihu, concentrations have been found to reach 217 ng·L^−1^ [4]. Similarly, BPA levels in the Yangtze River Basin have been detected with concentrations of 0.98 to 43.7 ng·L^−1^ [5]. These findings highlight the significant environmental impact of BPA and underscore the need for effective removal strategies.

Numerous effective methods are available for BPA degradation, including physical, chemical, and biological approaches. Techniques such as ozonation and photocatalytic, electrocatalytic, and persulfate oxidation are effective in degrading BPA. Advanced oxidation processes (AOPs) represent a powerful strategy for the degradation of organic pollutants such as BPA [6], with peroxymonosulfate (PMS) activation being a key technique for generating highly reactive species, including sulfate (SO_4_^•−^) and hydroxyl (•OH) radicals [7]. Redox-active transitional metal species, particularly iron (Fe), copper (Cu), and manganese (Mn) in various oxidation states, demonstrate exceptional persulfate-activating performance, owing to their electron-transfer capabilities [8]. Although transitional metal catalysts exhibit remarkable effectiveness in organic contaminant removal, two major technical challenges require further resolution: (1) inevitable catalyst deactivation during prolonged operation [9], and (2) the potential risk of toxic metal leaching into treated water systems. Therefore, to meet the stringent requirements of practical wastewater treatment applications, ideal persulfate-activating catalysts must exhibit four essential characteristics: (I) high catalytic activation efficiency, (II) long-term operational stability, (III) minimal environmental toxicity, and (IV) economic feasibility.

Layered double hydroxides (LDHs) have been extensively explored as high-efficiency catalysts in AOPs because of their unique structural and compositional advantages, such as flexible tunability, robust thermal stability, high density of active sites, and facile anion-exchange capability [10,11]. According to Ren [12], co-containing catalysts generally exhibit superior PMS activation capabilities compared to other transitional metal-based catalysts. Consequently, many researchers have used Co(II)-based LDHs for efficient PMS activation, such as Co(II)-intercalated NiFe-LDH [13], CoMn-LDH [14], and CoCu-LDH/PMS catalytic systems [15]. However, it is important to highlight that, even at trace concentrations, the leaching of cobalt ions from Co(II)-based LDHs can lead to significant biotoxicity and potential environmental hazards, thereby imposing a considerable limitation on their widespread utilization [16]. Given growing environmental concerns, research efforts have strategically transitioned toward developing less hazardous alternatives, particularly Cu(II) and Mn-intercalated LDHs, which demonstrate comparable catalytic efficacy and reduced ecotoxicological impacts [17]. Yan [18] successfully prepared a CuMgFe-LDH and used it to activate persulfate for effective ethylbenzene degradation. Similarly, MnFe-LDHs have also shown excellent catalytic performance for the degradation of organic dyes [19]. Yan et al. confirmed that MgMnFe-LDHs with an Mn/Mg ratio of 0.5 exhibited optimal PMS activation capability and superior catalysis performance, whereby 93.1% degradation of imidacloprid was achieved within 30 min under the condition of 250 mg·L^−1^ of LDH and 0.65 mM of PMS [20]. These results highlight the outstanding catalytic performance of Cu(II)- and Mn-based LDHs. Our research group has successfully synthesized Cu_3_Mn-LDH, which demonstrates superior efficiency in catalyzing BPA degradation [21]. However, during our series of studies on Cu_3_Mn-LDH, we discovered that the material still faces several issues, particularly activity decay after multiple cycles of use, metal leaching, and aggregation of the active sites. These challenges significantly compromise their long-term practical applicability, which remains an urgent issue that must be addressed.

**Figure 1:**
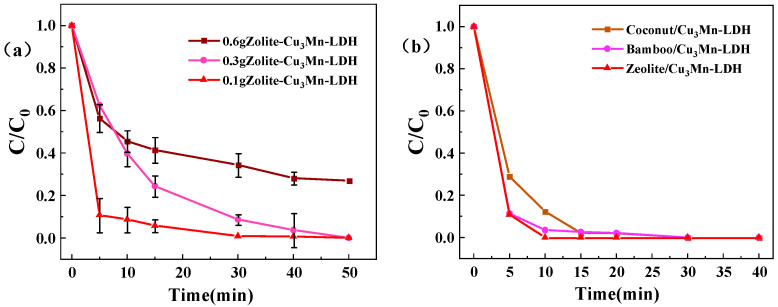
Effects of differing zeolite qualities on the BPA degradation performance of Z-LDH materials (**a**). Explain the situation of different loads (**b**).

The practical application of pristine Cu_3_Mn-LDH is constrained by its tendency to deactivate over multiple cycles, a phenomenon often linked to the aggregation of active sites and metal ion leaching. A promising strategy to mitigate these issues, as documented in the literature, is to support LDH nanomaterials on a stable matrix. Various materials, including activated carbon, graphene, and other molecular sieves, have been successfully employed to enhance the dispersion and stability of LDHs [22,23]. Guided by this principle, we aimed to identify an optimal, economical support for Cu_3_Mn-LDH. We initiated a comparative screening of three candidate supports: coconut biochar, bamboo biochar, and zeolite. The rationale for their selection was based on their proven adsorptive properties, high surface area, and potential to create synergistic interfaces with LDHs. The catalytic performance of the resulting composites—Coconut/Cu_3_Mn-LDH, Bamboo/Cu_3_Mn-LDH, and Zeolite/Cu_3_Mn-LDH (Z-LDH)—was systematically evaluated for BPA degradation. The results, presented in Figure 1b, unequivocally showed that Z-LDH achieved the highest degradation rate, nearing complete BPA removal within 20 min, and thus was identified as the optimal candidate. The exceptional efficacy of zeolite is likely derived from its intrinsically ordered microporous structure and high thermal stability. These properties facilitate a more uniform distribution of Cu_3_Mn-LDH platelets, prevent their restacking, and provide a confined environment that enhances the interfacial electron transfer during PMS activation, thereby boosting both activity and stability. Zeolites have emerged as promising materials for AOPs, owing to their unique structural and chemical properties. They have been extensively studied for their ability to catalyze the oxidation of Volatile Organic Compounds (VOCs) and offer several advantages such as high thermal stability, adjustable pore structure, and excellent adsorption capabilities [24]. Meanwhile, zeolite serves to minimize metal leaching, thus preventing the agglomeration of molecules [25,26]. When supported with metal catalysts, zeolites show significant advancements in catalytic applications, particularly in heterogeneous catalytic processes such as hydrogenation reactions and the oxidation of organic compounds [27]. In this study, a composite catalyst of Zeolite/Cu_3_Mn-LDH was synthesized via the co-precipitation method and used in the PMS activation system for BPA removal. The physicochemical properties of the catalyst were comprehensively characterized using various methods. The impacts of catalyst dosage, PMS concentration, and initial pH on BPA removal were assessed. Additionally, the mechanism and degradation pathways of BPA in the zeolite/Cu_3_Mn-LDH/PMS system were investigated based on the results of the quenching experiments, Electron Paramagnetic Resonance (EPR) analysis, transformation products, and structural characteristics of the catalyst after the catalytic reaction.

## 2. Experimental Section

### 2.1. Materials

The chemical reagents, of Cu(NO_3_)_2_·3H_2_O (99.9), Mn(NO_3_)_2_·4H_2_O solution (99.95), NaOH, and Na_2_CO_3_ are all analytically pure. Chemical samples were purchased from Xilong Scientific Co. Ltd. (Shantou, China), and all solutions were prepared using deionized water (DI). Zeolites were purchased from Ningbo Jiahe New Materials Co., Ltd. (Ningbo, China).

### 2.2. Cu_3_Mn-LDH and Zeolite/Cu_3_Mn-LDH(Z-LDH) Preparation

#### 2.2.1. Cu_3_Mn-LDH Preparation

Cu_3_Mn-LDH was successfully synthesized using the co-precipitation method as our research group reported [21].The specified amounts of Cu(NO_3_)_2_·3H_2_O (99.9%) and Mn(NO_3_)_2_·4H_2_O (99.95%) were dissolved in deionized water. The mixed metal solution was kept at a constant temperature of 60 ± 0.5 °C with vigorous stirring (800 rpm) and the pH was precisely titrated to 10.0 ± 0.2 using 1M NaOH. Controlled oxidation was achieved through incremental H_2_O_2_ (30% *w*/*w*) addition until the characteristic blue coloration dissipated (indicating complete Mn^2+^ → Mn^3+^ oxidation). The colloidal product was aged for 12 h at 60 °C, then centrifuged, washed with deionized water, and final vacuum-dried at 80 °C for 24 h. A systematic variation in the Cu:Mn feeding ratio allowed for precise control over the composition of resulting material, with a 3:1 ratio showing optimal PMS activation efficiency; thus, it was selected for subsequent modification studies.

#### 2.2.2. Z-LDH Preparation

In a 1000 mL beaker, 10.128 g of Cu(NO_3_)_2_·3H_2_O (99.9) was dissolved in 300 mL of deionized water. To this solution, 4.2 mL of an 80% Mn(NO_3_)_2_·4H_2_O solution (99.95) solution was added, followed by the addition of varying amounts (0.1, 0.3, and 0.6 g) of 200-mesh zeolite. We dissolved 10.128 g of Cu(NO_3_)_2_ in 300 mL of DI in a 1000 mL beaker. The mixture was stirred at 60 °C and 500 mL of a pH-regulating solution was gradually added, prepared by dissolving 4g of NaOH and 2g of Na_2_CO_3_, until the pH reached approximately 10. Subsequently, an appropriate amount of H_2_O_2_ was added until the solution became colorless. The resulting mixture was agitated at 80 °C for 40 h. After aging, the mixture was filtered and the product was washed DI until a neutral pH was achieved. The product was centrifuged and dried at 105 °C until a constant weight was achieved to obtain Z-LDH. When synthesizing the material, zeolite (0.1 g, 0.3 g and 0.6 g) was added, and the composite with the highest degradation efficiency was selected for further experiments.

### 2.3. Analysis and Characterization Methods

The synthesized materials were characterized using a suite of techniques to determine their crystallinity, functional groups, morphology, elemental composition, and surface properties. X-ray diffraction (XRD) was performed on a Netherlands X’Pert3 Power diffractometer (PNAlytical B.V., Almelo, The Netherlands) using Cu Kα radiation (λ = 1.5406 Å) to identify the crystalline phases present. Fourier-transform infrared (FT-IR) spectroscopy was carried out on a Thermo Scientific iN10 spectrometer (Perkin Elmer, Waltham, MA, USA) in the range of 4000–500 cm^−1^ to detect specific functional groups and chemical bonds. The material’s morphology and microstructure were examined by field emission scanning electron microscopy (FE-SEM) on a JSM-7900F instrument (Shengze Technology, Guangdong, China) and by transmission electron microscopy (TEM) on a JEOL JEM 2100F microscope (JEOL Ltd., Tokyo, Japan), with samples prepared by dispersing the powder in ethanol and depositing it on a carbon-coated copper grid. Surface elemental composition and chemical states were analyzed by X-ray photoelectron spectroscopy (XPS) using a Thermo Scientific K-Alpha instrument (Thermo Fisher, Waltham, MA, USA) with an Al Kα X-ray source. The specific surface area and pore size distribution were determined from N_2_ adsorption–desorption isotherms measured at 77 K using a Micromeritics ASAP2020M+C analyzer (Micromeritics, Norcross, GA, USA), with data processed by the Brunauer–Emmett–Teller (BET) and Barrett–Joyner–Halenda (BJH) methods. Particle size and zeta potential were measured using a Nano ZS 90 analyzer (Malvern Panalytical, Malvern, UK) to assess the colloidal stability of the materials in aqueous suspension. Inductively coupled plasma optical emission spectrometry (ICP-OES) (Perkin Elmer, Waltham, MA, USA) was employed to quantify metal content and leaching after digesting the samples in acid. Finally, the concentration of BPA during degradation experiments was monitored by high-performance liquid chromatography (HPLC) using an Agilent 1260 system (Agilent, Santa Clara, CA, USA) equipped with a C18 column and a UV detector (Agilent, Santa Clara, CA, USA).

### 2.4. Evaluation of Catalytic Performance: BPA Degradation and Reaction Kinetics

The batch degradation experiments were systematically conducted in 500 mL glass bottles, with continuous stirring facilitated by a magnetic stirrer, to assess various operational parameters, including: (i) catalyst dosage (0.025–0.2 g·L^−1^), (ii) oxidant concentration (PMS, 0.15–0.3 g·L^−1^), (iii) pollutant concentration (BPA, 5–30 mg·L^−1^), (iv) coexisting anions effects (0.4 mM Cl^−^, 0.4 mM HCO_3_^−^, 0.4 mM H_2_PO_4_^−^, 1 mg·L^−1^ HA), (v) temperature dependence (15–45 °C), (vi) pH influence, (vii) mineralization efficiency (total organic carbon (TOC) analysis), and (viii) catalyst reusability. After the predetermined reaction time, samples were taken from the reaction solution with a syringe, filtered through a 0.45 µm water membrane, and transferred to sample bottles. Quantitative analysis of BPA was performed by reversed-phase HPLC(Agilent 1260) using a Phenomenex^®^ RX-C18 analytical column (4.6 × 250 mm length, 5 μm particle size) (Agilent, Santa Clara, CA, USA). The isocratic elution was conducted with a methanol (MeOH)/water (50:50, *v*/*v*) mobile phase at a flow rate of 1.0 mL·min^−1^, with Ultraviolet (UV) detection set at 276 nm.

### 2.5. Reactive Species Identification

To identify the reactive oxygen species (ROS) responsible for BPA degradation by the Z-LDH+PMS system, radical quenching tests and electron paramagnetic resonance (EPR) analysis was employed. To quantitatively evaluate the distinct roles of (SO_4_^•−^), (•OH), superoxide radicals (O_2_•^−^), and singlet oxygen (^1^O_2_), specific scavengers—including MeOH (250 mM·L^−1^), tert-butanol (TBA) (250 mM·L^−1^), p-BQ (10 mM·L^−1^), and NaN_3_ (10 mM·L^−1^) —were employed to selectively quench each reactive species. To further confirm the types of radicals, complementary EPR analysis using (5,5-dimethyl-1-pyrroline N-oxide)(DMPO) and (2,2,6,6-tetramethylpiperidine N-oxide)(TEMP) spin-trapping agents was performed, allowing for the direct detection and identification of the specific radicals produced in the Z-LDH+PMS system.

## 3. Results and Discussion

### 3.1. Material Stoichiometry Optimization

During synthesis of the materials, zeolite powder (0.1, 0.3, 0.6 g; 200 mesh) was added to the material precursor solution during the hydrothermal co-precipitation process. The three prepared materials were then tested for catalytic PMS BPA degradation, with an initial BPA concentration of 10 mg·L^−1^. As illustrated in Figure 1a, the material with a zeolite loading of 0.1 g demonstrated the highest degradation efficiency, outperforming other compositions with different zeolite ratios. This indicates that optimal zeolite loading is crucial for enhancing the catalytic performance of CuMn-LDH materials in BPA degradation. Based on these results, the Z-LDH synthesized with 0.1 g of zeolite was selected for further investigation in subsequent experiments, ensuring that the performance of material was optimized for practical applications.

The superior performance at the 0.1 g zeolite loading can be rationalized by an optimal balance between several factors. First, at this loading, the zeolite achieves effective dispersion on the Cu_3_Mn-LDH framework, maximizing the creation of synergistic interfaces. The strategy of constructing a synergistic interface between an adsorbent and a catalyst to enhance reaction kinetics is well-established [28]. Furthermore, the zeolite particles act as structural stabilizers, helping to prevent the restacking of LDH layers and maintain accessibility, a principle demonstrated in other composite material systems [29]. Conversely, higher loadings result in excessive zeolite coating, which is known to cause active site blockage and mass transfer limitations [30], explaining the observed decline in activity.

Coconut shell biochar, bamboo biochar, and zeolite were selected as carriers for supporting Cu_3_Mn-LDHs. To identify the optimal material, the PMS activation performance of Coconut/Cu_3_Mn-LDH, Bamboo/Cu_3_Mn-LDH, Z-LDH, and pure Cu_3_Mn-LDH in degrading BPA was evaluated, with the results shown in Figure 1b. The composite materials exhibited significantly enhanced reaction rates compared to pure Cu_3_Mn-LDH [21], with the order of degradation efficiency being Z-LDH > Bamboo/Cu_3_Mn-LDH > Coconut/Cu_3_Mn-LDH. Z-LDH achieved nearly complete degradation of BPA within 20 min. Therefore, Z-LDH was selected as the subject material for further investigation.

### 3.2. Z-LDH Catalysts Characterization

#### 3.2.1. XRD Analysis

**Figure 2:**
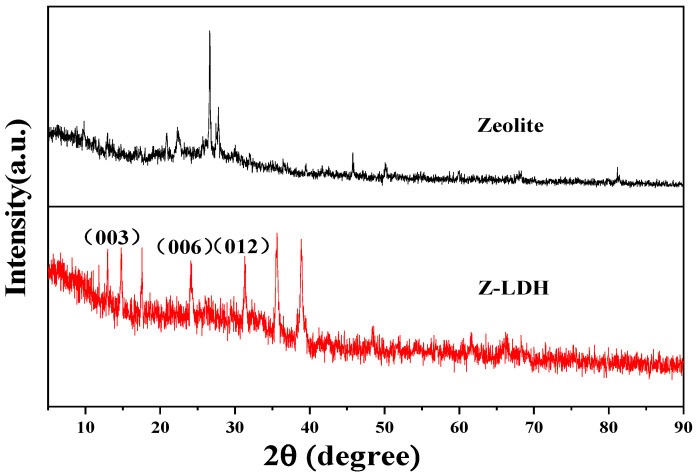
Zeolite and Z-LDH XRD patterns.

X-ray diffraction (XRD) analysis was performed to determine the crystallographic structure, phase purity, and successful formation of the Z-LDH composite. XRD analysis was performed to investigate the Z-LDH structural properties. The XRD patterns of both the zeolite and Z-LDH were obtained (Figure 2). For zeolite, the diffraction peaks at 2θ = 21°, 24.5°, 36.7°, 39.5°, 46.1°, and 50° corresponded to the (101), (110), (102), (201), and (112) PDF#46-1045 planes, respectively. Peaks at 2θ = 23.15° and 27.4° corresponded to the (122) and (400) PDF#35-0344 planes, respectively. The additional peaks at 2θ = 26.8°, 45.7°, and 68.2° corresponded to the (111), (131), and (202) PDF#33-1200 planes. These diffraction patterns indicate that the materials in the zeolite are primarily composed of SiO_2_, Na_4_SiO_4_, and NaAlO_2_, confirming that the zeolite is of the orthorhombic type [31]. When zeolite was combined with Cu_3_Mn-LDH, the characteristic LDH peaks, such as (003), (006), and (012), remained present, indicating that the hydrotalcite structure was retained [21]. Additionally, several new peaks appeared at 2θ = 14.7°, 17.5°, and 38.8°, corresponding to the (020), (120), and (150) PDF#35-0481 planes, respectively. These results suggest that the zeolite successfully combined with Cu_3_Mn-LDH, forming a more compact and complex crystal structure with improved crystallinity. However, the characteristic zeolite peaks were not observed in the Z-LDH XRD pattern. This could be due to the interaction between the loading material and the zeolite, which possibly decreased the crystallinity of the zeolite, leading to the disappearance or shifting of the characteristic infrared peaks and affecting the display of the XRD peaks. Additionally, the loading material may block certain zeolite pores, reducing their micropore volume, which could explain the absence of some characteristic XRD peaks [32,33]. This phenomenon indicates that the zeolite has been successfully loaded into the pores of the Cu_3_Mn-LDH precursor, forming stable nanoclusters that influence the crystallinity of the zeolite and affect the XRD results. The absorption peak in the 30–40 cm^−1^ range observed during the transformation from the precursor to Z-LDH offers further evidence of the phase transition in the synthesized hybrid material [34].

#### 3.2.2. Zeolite FTIR Spectra

**Figure 3:**
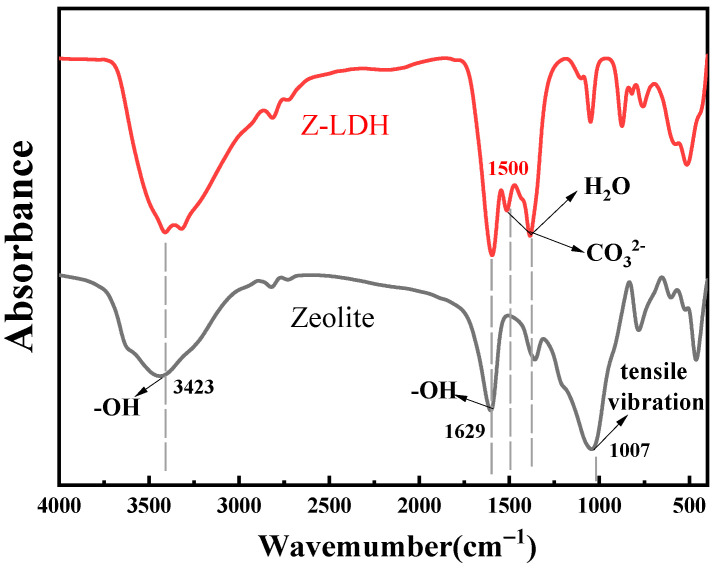
FTIR spectra of zeolite and Z-LDH.

The zeolite and Z-LDH FTIR spectra were recorded to examine the surface functional groups and bonding interactions, and the results are shown in Figure 3. It is evident that, after the modification of Cu_3_Mn-LDH, the Z-LDH absorption peak at 1500 cm^−1^ experiences a significant shift. Similarly to pure Cu_3_Mn-LDH, all the characteristic LDH peaks were observed for Z-LDH. The Z-LDH absorption peaks at 1420 and 1353 cm^−1^ originated from adsorbed water and the Cu_3_Mn-LDH CO_3_^2−^ interlayer, respectively. The weak band observed at 861 cm^−1^ corresponds possibly due to Mn-OH stretching vibration. Furthermore, several absorption bands observed in the 500–900 cm^−1^ region were assigned to vibrational modes involving metal-oxygen bonds, specifically Cu-O and Mn-O stretching vibrations, as well as the translational modes of M-O-M and O-M-O bridges (where M = Cu, Mn) in the structure [15]. The characteristic zeolite peaks were also observed for Z-LDH. The bands observed between 1629 and 1637 cm^−1^ and the broad band at approximately 3423–3438 cm^−1^ in Z-LDH were attributed to the deformation and stretching vibrations of the OH groups in water molecules. The absorption peak at 1007.4 cm^−1^ corresponds to the asymmetric stretching vibration of polyhedral structures within the material [35]. Consequently, after the zeolite was loaded with Cu_3_Mn-LDH, the peak shapes observed for Z-LDH matched those of the individual components, suggesting that zeolite composites with Cu_3_Mn-LDH were embedded. The incorporation of zeolite improved the surface functional group structure of Cu_3_Mn-LDH and enriched the functional groups in Z-LDH, which is beneficial for catalyzing PMS reactions to degrade BPA.

#### 3.2.3. SEM and TEM Analysis

**Figure 4:**
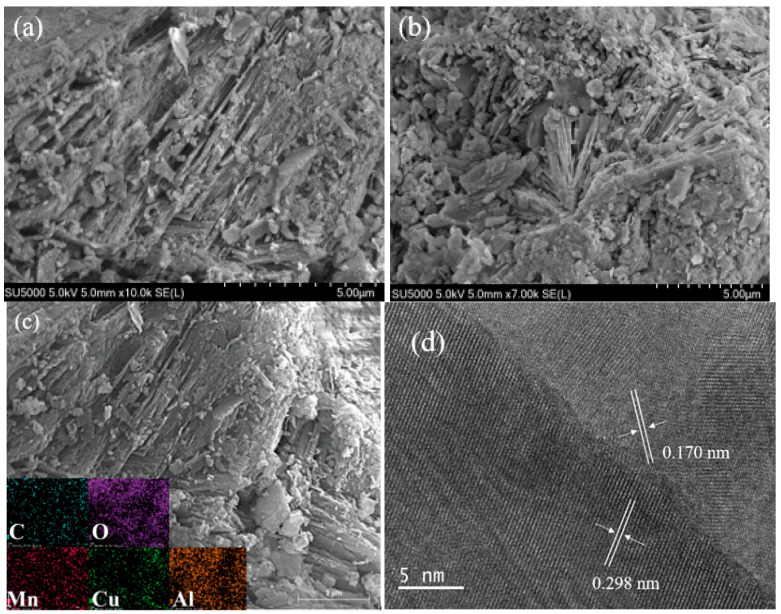
SEM image of zeolite material (**a**), SEM image of Z-LDH material (**b**), EDS image of Z-LDHs (**c**), and HR-TEM image of Z-LDH (**d**).

Figure 4a,b display the SEM images of the Z-LDH and zeolite materials, respectively. Figure 4a shows the characteristic morphology of the pristine zeolite material, displaying well-defined band-like structures with slanted textures that constitute the typical layered framework of zeolitic materials [36]. In contrast, Figure 4b shows the modified morphology of the Z-LDH composite, where the zeolite surface is decorated with uniformly distributed plate-like Cu_3_Mn-LDH nanostructures [21]. The distinct structural evolution from the pristine zeolite to the composite material demonstrates successful heterostructural formation, indicating that the zeolite framework effectively functions as a structural support for Cu_3_Mn-LDH nucleation and growth. Furthermore, the intercalation of Cu_3_Mn-LDH into the zeolite pores was confirmed through a comparative morphological analysis [37,38]. The elemental distribution across Z-LDH composite were further verified by energy dispersive spectroscopy(EDS) mapping, as shows in Figure 4c. The presence of zeolite is evident from the significant distribution of aluminum (Al) on the elemental map, with a uniform distribution across the sample. This indicates that the composite formed by the zeolite and Cu_3_Mn-LDH is homogeneous with uniform incorporation [39]. The elemental composition analysis presented in Figure S1 reveals the atomic percentages of the constituent elements in Z-LDH as: carbon (C) (21.73%), oxygen (O) (58.26%), (Cu) (3.22%), (Mn) (2.72%), and Al (4.08%). This quantitative elemental distribution confirms the successful incorporation of both transitional metals (Cu, and Mn) and framework elements (C, O, and Al) into the composite material. High-resolution TEM analysis of Z-LDH (Figure 4d) shows well-defined lattice fringes with interplanar spacings of 0.170 and 0.298 nm, corresponding to the (120) crystallographic plane of NaAlO_2_ (PDF# 33-1200) and the (002)/(170) planes of (Mg,Cu^2+^)_2_(CO_3_)(OH)_2_ (PDF# 35-0481), respectively [40]. Microstructural characterization clearly demonstrates the formation of distinct phase boundaries between the zeolite frameworks [41]. And Cu_3_Mn-LDH components. These interfacial heterojunctions are particularly significant because they provide efficient electron transfer pathways during redox processes, thereby enhancing PMS activation and subsequently improving BPA degradation efficiency. Representative TEM images of Z-LDH are shown in Figure S2.

#### 3.2.4. Specific Surface Area Analysis and Zeta Potential

**Figure 5:**
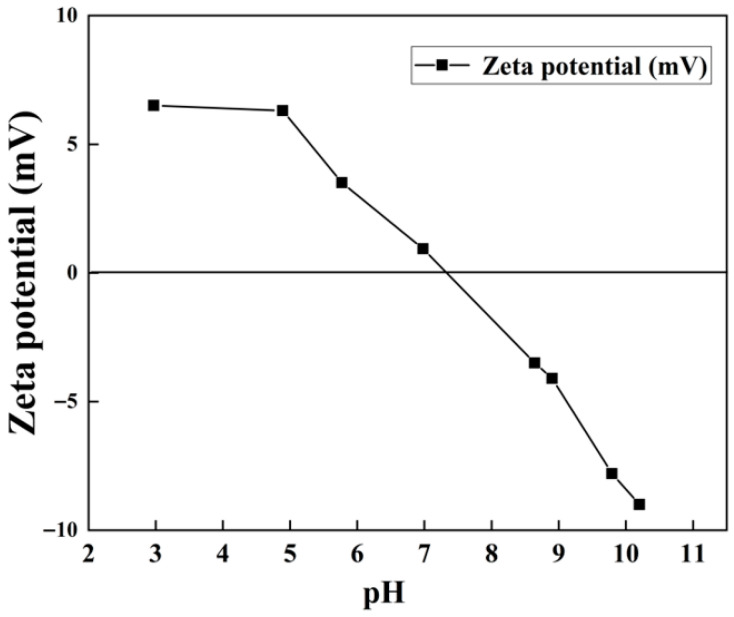
Zeta potential of Z-LDH.

As illustrated shown in Figure 5, the zeta potential variation trend of Z-LDH exhibits a pattern similar to that of pristine LDH [21], with the surface becoming progressively more negatively charged as increase pH values. The pH pH_pzc_ of Z-LDH was measured as 7.35. Notably, a comparative analysis revealed that the zeta potential values of Z-LDH are substantially lower than those of Cu_3_Mn-LDH. This significant reduction originated primarily from the intrinsic negative surface charge of the incorporated zeolite component, thereby effectively modulating the overall surface charge characteristics of the composite material. Importantly, Z-LDH maintained a broad pH applicability range comparable to that of Cu_3_Mn-LDH, while demonstrating superior degradation performance. This enhanced catalytic activity suggested that the modified zeta potential profile of Z-LDH had a beneficial effect on its functional properties.

As summarized in Table S1, the Brunauer–Emmett–Teller analysis revealed that zeolite, Cu_3_Mn-LDH, and Z-LDH exhibited specific surface areas of 36.46, 101.25 [21], and 122.72 m^2^·g^−1^, respectively. Although zeolite alone exhibited the lowest specific surface area, the composite Z-LDH demonstrated a significant enhancement (122.72 m^2^·g^−1^) compared to those of both pristine zeolite and Cu_3_Mn-LDH. This notable surface area enhancement originates from the successful integration of zeolite into the Cu_3_Mn-LDH matrix, which possibly creates additional porous structures and thereby increasing the accessibility of active sites. The enlarged surface area following composite formation is critical for improving the catalytic performance. In PMS based advanced oxidation systems, the reaction between metal ions and PMS occurs via solid surface of the catalyst, exhibiting strongly dependent on available surface area [42,43]. Specifically, the expanded interfacial contact area between the catalyst and PMS in solution facilitates a more efficient electron transfer, thereby enhancing catalytic oxidation kinetics [44,45]. This structural advantage not only promoted the generation of ROS but also significantly accelerated the BPA degradation efficiency [45]. Figure S3 (Supporting Information) presents the N_2_ adsorption–desorption isotherms of the Z-LDH composite.

### 3.3. BPA Catalytic Oxidation in Z-LDH+PMS

#### 3.3.1. Effect of Materials Dosage

**Figure 6:**
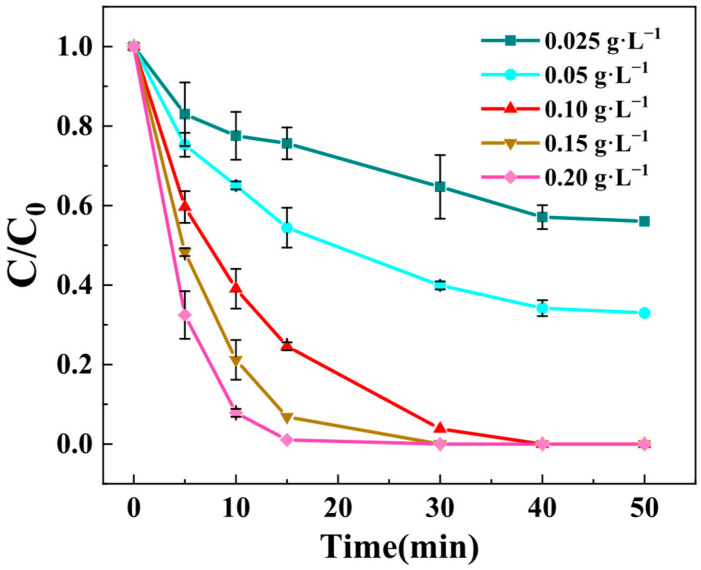
Effects of Z-LDH dosage on BPA degradation rates. (Experimental conditions: PMS concentration = 0.15 g⋅L^−1^, BPA concentration = 10mg⋅L^−1^, temperature = 25 °C, initial pH = 7.0 ± 0.1).

The influence of Z-LDH dosage on BPA degradation was systematically investigated in the Z-LDH/PMS system. The experimental results demonstrated distinct dosage-dependent catalytic behavior. As illustrated in Figure 6, when employing a minimal Z-LDH dosage of 0.025 g·L^−1^, the catalytic system demonstrated constrained oxidative capacity, attaining merely 40% BPA degradation efficiency following a 50 min reaction period. The degradation efficiency correlated positively with catalyst loading, reaching a substantial enhancement at 0.10 g·L^−1^. While, further dosage increase to 0.20 g·L^−1^ caused adverse effects, suggesting the attainment of optimal catalytic conditions. Based on these observations, the optimal Z-LDH dosage range for BPA degradation was determined as 0.15–0.20 g·L^−1^. Consequently, a moderate dosage of 0.15 g·L^−1^ was chosen as for subsequent experiments to balance performance and cost-effectiveness.

The catalytic performance of Z-LDH was strongly dependent on its dosage, which can be directly correlated with its enhanced specific surface area and optimized surface charge properties as revealed by BET and zeta potential analyses (Section 3.2.4). The composite exhibited a BET surface area of 122.72 m^2^·g^−1^, significantly higher than that of zeolite (36.46 m^2^·g^−1^). This increased surface area provided more active sites for PMS activation and BPA adsorption. As shown in Figure 6, a dosage of 0.10 g·L^−1^ achieved nearly complete BPA degradation within 20 min, while lower dosages (e.g., 0.025 g·L^−1^) resulted in insufficient active sites, leading to only 40% degradation. However, excessive dosage (0.20 g·L^−1^) did not further enhance the degradation rate, likely due to agglomeration or overlapping of active sites, as suggested by the SEM and TEM images (Figure 4), which showed uniform but dense distribution of Cu_3_Mn-LDH on zeolite.

#### 3.3.2. Effect of PMS Concentration

**Figure 7:**
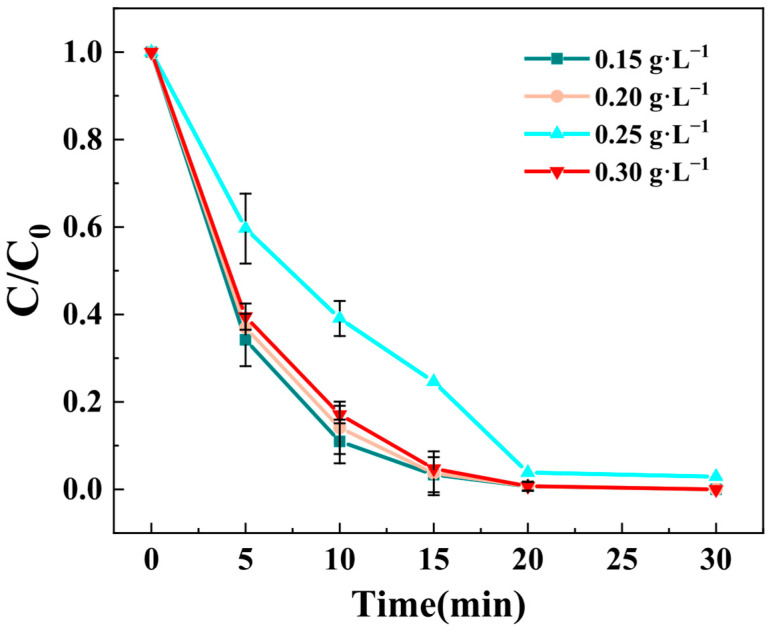
Effect of PMS dosage on BPA removal by Z-LDH. (Experimental conditions: Z-LDH concentration = 0.15g⋅L^−1^, BPA concentration = 10mg⋅L^−1^, temperature = 25 °C, initial pH = 7.0 ± 0.1).

The catalytic system consists of three essential components: BPA as the target pollutant, Z-LDH as the heterogeneous catalyst, and PMS as the oxidant precursor. Among these, PMS serves as the key driver in the AOP by serving as the primary source for ROS generation. As displayed in Figure 7, the catalytic degradation performance of BPA was strongly influenced by PMS dosage varied from 0.15 to 0.3 g·L^−1^. The degradation efficiency at a PMS concentration of 0.25g·L^−1^ is markedly lower than at other concentrations, whereas the efficiency does not exhibit much variation much between 0.15 and 0.2 g·L^−1^. The experimental results demonstrate a non-linear relationship between PMS dosage and degradation efficiency. At 0.25 g·L^−1^ PMS concentration, the system shows markedly inferior BPA removal efficiency compared to other tested concentrations. Interestingly, minimal variation in BPA removal efficiency was obtained within the 0.15–0.2 g·L^−1^ range. Notably, the maximum degradation BPA removal efficiency was not achieved at the highest PMS dosage (0.3 g·L^−1^), suggesting an optimal concentration threshold. This phenomenon can be attributed to the competitive radical reactions occurring at elevated PMS concentrations. Excessive PMS leads to [46,47]: (1) self-scavenging effects between SO_4_^•^ and •OH; (2) unproductive consumption of generated ROS; and (3) Incomplete utilization of the oxidant. The optimal degradation performance was achieved at 0.15 g·L^−1^ PMS concentration, where the Z-LDH catalyst demonstrated maximum activation efficiency. This observation confirms that the catalytic system requires precise PMS dosage control to balance the radical generation and consumption processes.

The Z-LDH catalyst exhibited excellent performance across a wide pH range (3.5–11), which can be attributed to its modulated surface charge and structural stability conferred by the zeolite support. The point of zero charge (pH_pzc_) of Z-LDH was 7.35 (Figure 5), indicating a more negatively charged surface that favors PMS anion (HSO_5_^−^) adsorption under neutral and alkaline conditions. This is further supported by XRD and FTIR results (Section 3.2.1 and Section 3.2.2), which confirmed the retention of the LDH structure and the presence of functional groups (e.g., -OH, CO_3_^2−^) that facilitate PMS activation.

#### 3.3.3. Effect of BPA Concentration

**Figure 8:**
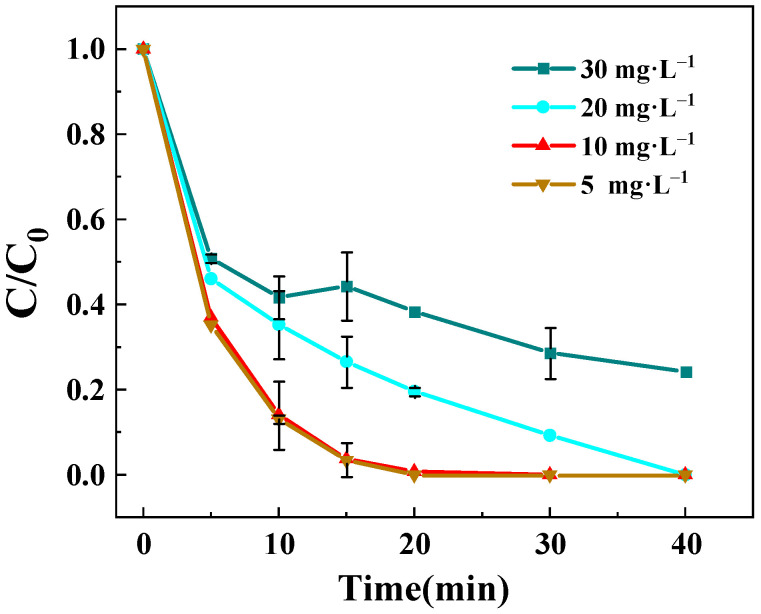
Effect of initial BPA solution concentration on the degradation rate of BPA degraded by Z-LDH. (Experimental conditions: Z-LDH concentration = 0.15 g⋅L^−1^, PMS concentration = 0.15 g⋅L^−1^, temperature = 25 °C, initial pH = 7.0 ± 0.1).

To elucidate the influence of BPA concentration on degradation kinetics, a series of experiments was performed at four different initial concentrations (5, 10, 20, and 30 mg·L^−1^), selected based on the aqueous solubility limit of BPA. As shown in Figure 8, the degradation efficiency is strongly dependent on the initial BPA concentration. The fastest degradation kinetics were observed at 5 mg·L^−1^, achieving complete removal within 15 min, whereas progressively slower degradation rates were recorded with increasing BPA concentrations. This concentration-dependent behavior can be attributed to the finite generation capacity of ROS by the Z-LDH+PMS system in a 400 mL reaction volume per unit of time. At the lowest concentration (5 mg·L^−1^), the instantaneous ROS production sufficiently met the oxidative demand for complete BPA mineralization. However, at elevated concentrations (20–30 mg·L^−1^), the substantial increase in BPA molecules necessitated sustained ROS generation to accomplish complete degradation. Numerous experimental results demonstrated that BPA concentration had a significant impact on the experiment, with different concentrations leading to varying reaction rates and degradation pathways during the degradation process [48]. Given the differing BPA concentrations, the reactivity also varied considerably [49]. At low BPA concentrations, complete degradation occurred within a short period of time. However, as BPA concentration increased, the rate of ROS production in the system did not meet the demand for BPA degradation, leading to a noticeable significant decline in BPA degradation rate [50]. Consequently, an intermediate concentration of 10 mg·L^−1^ was adopted as subsequent investigations as it represented an optimal balance between reaction kinetics and practical applicability.

#### 3.3.4. Effect of Coexisting Anions

**Figure 9:**
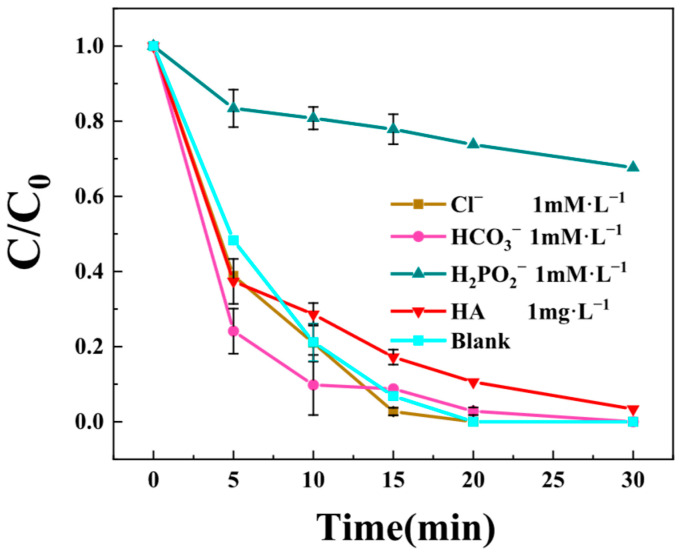
Influence of coexisting anions on BPA degradation efficiency in the Z-LDH+PMS system. (Experimental conditions: Z-LDH concentration = 0.15 g⋅L^−1^, PMS concentration = 0.15g⋅L^−1^, BPA concentration = 10mg⋅L^−1^, temperature = 25 °C, initial pH = 7.0 ± 0.1).

The impacts of various anions (Cl^−^, HCO_3_^−^, H_2_PO_4_^−^ and humic acid)HA on BPA degradation efficiency were systematically investigated in the Z-LDH+PMS system. As illustrated in Figure 9, the presence of Cl^−^, HCO_3_^−^, and HA exhibited adverse impact on BPA removal by Z-LDH+PMS system, with HCO_3_^−^ demonstrating a notable promotional effect. Specifically, BPA removal efficiency was enhanced by approximately 25% within the initial 15 min when HCO_3_^−^ was present. In contrast, the addition of introduction H_2_PO_4_^−^ caused a dramatic 70% decrease in BPA degradation efficiency, indicating a strong inhibitory effects.

The different effects of these anions can be attributed to their selective interactions with the catalytic system [51]. Three key observations merit discussion: First, both Cl^−^ and HA showed minimal interference with the reaction kinetics. This phenomenon may be explained by the superior structural characteristics of Z-LDH compared to pristine Cu_3_Mn-LDH materials [21]. Specifically, the Z-LDH composite possesses: (1) a more diverse array of surface functional groups, (2) an expanded specific surface area, and (3) enhanced metal dispersion (Al, and Ca). These properties facilitate effective Cl^−^ and HA adsorption onto the catalyst surface, thereby preventing radical scavenging by Cl^−^.

Second, HCO_3_^−^ exhibited a unique promotional effect, which can be explained by two synergistic mechanisms: (1) the inherent presence of HCO_3_^−^ and CO_3_^2−^ as interlayer anions in the Z-LDH structure creates favorable interaction sites, and (2) additional HCO_3_^−^ in solution undergoes selective adsorption onto zeolitic surfaces, promoting PMS activation through surface-mediated electron transfer processes.

Most significantly, H_2_PO_4_^−^ displayed pronounced inhibition (70% efficiency loss), which is consistent with previous reports on Cu_3_Mn-LDH+ PMS systems [21]. This strong inhibitory effect stems from the formation of stable Cu-HPO_4_^2−^ complexes that deactivate the catalytic sites, effectively blocking PMS activation and subsequent BPA degradation [52].

The resistance of Z-LDH to common anions (Cl^−^, HCO_3_^−^) and humic acid can be linked to its hierarchical pore structure and enhanced metal dispersion, as observed in SEM-EDS and BET analyses. The uniform distribution of Al and Cu (Figure 4c) and the high surface area likely provided abundant adsorption sites, minimizing the scavenging of radicals by Cl^−^. In contrast, the strong inhibition by H_2_PO_4_^−^ is consistent with the formation of stable Cu–HPO_4_ complexes, which block active sites, as also evidenced by the XPS analysis of Cu species.

#### 3.3.5. Effect of Solution Temperature

**Figure 10:**
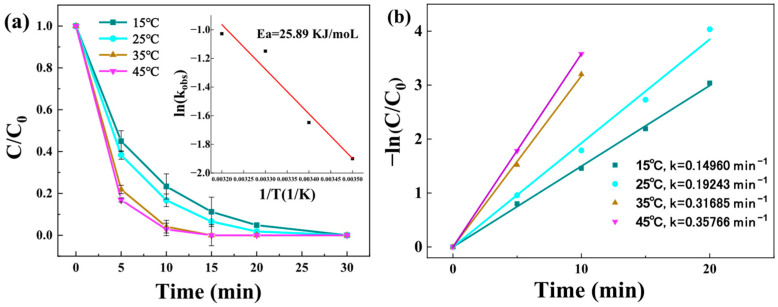
Influence of temperature on BPA degradation by Z-LDH (**a**) (The red line represents the relationship between the chemical reaction rate constant of Z-LDH and temperature, as obtained from the Arrhenius equation.), the pseudo-first-order kinetic regression curve of BPA degradation (**b**). (Experimental conditions: Z-LDH concentration = 0.15g⋅L^−1^, PMS concentration = 0.15 g⋅L^−1^, BPA concentration = 10mg⋅L^−1^, initial pH = 7.0 ± 0.1).

Environmental temperature was identified as a critical parameter influencing degradation kinetics. As demonstrated in Figure 10a, the BPA degradation efficiency exhibited strong temperature dependence, with complete pollutant removal achieved within 10 min at 45 °C. This remarkable performance can be attributed to two synergistic mechanisms: (1) thermally enhanced PMS self-activation to generate ROS, and (2) accelerated molecular collisions at elevated temperatures that promote radical generation and subsequent oxidation reactions. The reaction kinetics were quantitatively analyzed using the Arrhenius equation, revealing an activation energy (Ea) of 25.89 kJ·mol^−1^. This value significantly exceeded the typical range for diffusion-controlled processes (10–13 kJ·mol^−1^) [53]. This confirms that the reaction rate is governed by surface chemical kinetics rather than by mass transfer limitations. The relatively high Ea value suggests a substantial energy requirement for the oxidation process, consistent with previous observations for Cu_3_Mn-LDH+PMS systems [21]. Moreover, pseudo-first-order kinetic analysis (Figure 10b) provided further mechanistic insights. The calculated rate constant (k = 0.35766 min^−1^ at 45 °C) was more than double that observed at 45 °C (k = 0.15 min^−1^), demonstrating the pronounced thermal activation effect on PMS decomposition. This temperature-dependent behavior follows the general trend observed in AOPs, where elevated temperatures typically enhance both radical generation and pollutant degradation rates.

#### 3.3.6. Impact of Initial pH

**Figure 11:**
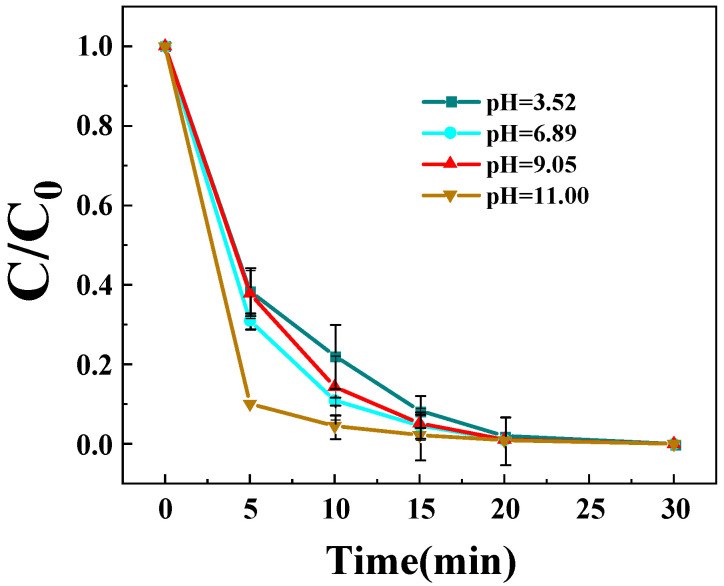
Effects of different pH on the degradation rate of BPA by Z-LDH. (Experimental conditions: Z-LDH concentration = 0.15 g⋅L^−1^, PMS concentration = 0.15 g⋅L^−1^, BPA concentration = 10 mg⋅L^−1^, temperature = 25 °C).

The solution pH significantly governs both the catalytic performance and reaction mechanism of the Z-LDH/PMS system, as evidenced in Figure 11. The degradation efficiency of BPA exhibited a clear pH-dependent trend, with significantly enhanced reaction kinetics observed under neutral to alkaline conditions. While complete BPA degradation required 20 min under strongly acidic conditions (pH = 3.52), it was achieved within only 15 min at near-neutral pH (6.89) This behavior can be illustrated by the pH-dependent PMS activation pathways. Under acidic conditions, the surface of Z-LDH becomes highly protonated, which hinders the effective adsorption and decomposition of PMS anions (HSO_5_^−^), leading to suppressed generation of sulfate radicals (SO_4_^•−^) as the primary reactive species, consistent with observations in other metal oxide systems [54]. In contrast, under neutral and alkaline conditions, the catalyst surface is more favorable for PMS activation. Notably, the Z-LDH/PMS system maintained high efficiency even at pH 11, unlike many conventional LDHs that suffer from metal hydroxide precipitation or decreased activity at high pH [55]. This suggests that the unique zeolite-modified structure not only enhances structural stability but also modulates the electron transfer pathway, potentially facilitating a non-radical dominant process (e.g., singlet oxygen generation or direct electron transfer) under alkaline conditions, a phenomenon recently highlighted in studies on modulated electron structures of catalysts [54,56]. The protective zeolite framework thus enables effective PMS activation across a wide pH range, representing a significant advancement in designing robust LDH-based systems for practical applications.

#### 3.3.7. Mineralization Performance: TOC Analysis

**Figure 12:**
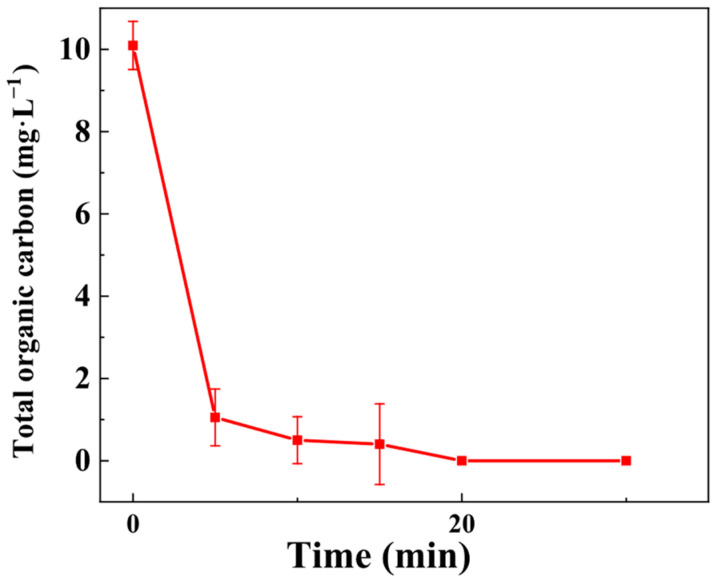
TOC removal of BPA degraded by Z-LDH+PMS. (Experimental conditions: Z-LDH concentration = 0.15 g⋅L^−1^, PMS concentration = 0.15 mg⋅L^−1^, BPA concentration = 10 mg⋅L^−1^, temperature = 25 °C, initial pH = 7.0 ± 0.1).

To fully characterize the degradation pathway, BPA elimination and mineralization efficiency were systematically evaluated. Time-dependent sampling was implemented, followed by TOC analysis to quantify the extent of organic pollutant mineralization. As shown in Figure 12, the TOC concentration decreased progressively with increasing reaction time, reaching near-zero levels after 20 min, corresponding to an exceptional TOC removal efficiency of 97.0%. This near-complete mineralization indicates that the Z-LDH/PMS system not only efficiently decomposed BPA into intermediate species, but also subsequently oxidized these fragments into environmentally benign end products, predominantly H_2_O and CO_2_. This exceptional mineralization efficiency underscores the capability of the system to minimize the accumulation of toxic byproducts, addressing a significant limitation commonly associated with conventional AOPs.

For example, previous studies using FeNi-LDH reported a TOC degradation rate of approximately 60% [57]. Notably, in the existing literature, the Fe-LDH membrane/PMS system demonstrated exceptional performance, achieving 89.6% TOC removal under a notably short retention time [58]. In comparison, our study yielded highly promising results, exhibiting a remarkably high TOC removal efficiency. Collectively, these findings highlight the potential of our experimental material as a promising way for BPA removal, thereby mitigating its environmental impact.

#### 3.3.8. Recycling Performance of the Material

**Figure 13:**
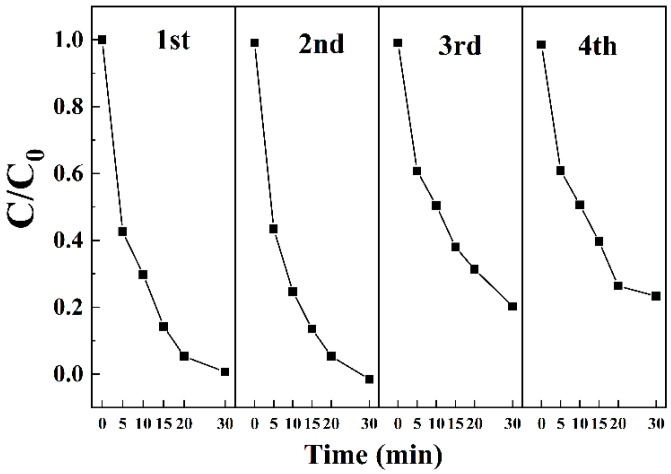
The effect of repetition on BPA degradation.

The cycling stability and practical reusability of Z-LDH as a heterogeneous catalyst were systematically evaluated for the PMS-activated oxidative degradation of BPA. As shown in Figure 13, the catalytic performance displays notable cycle-dependent behavior, characterized by progressive efficiency attenuation during successive reuse tests. The system initially demonstrated exceptional catalytic activity, degrading 10 mg·L^−1^ BPA completely within 30 min under standard reaction conditions. However, performance degradation became evident upon repeated cycling, with the efficiency decreasing to approximately 80% after three consecutive operational cycles under identical conditions. This observed performance decay possibly stems from multiple interrelated factors, including the accumulation of persistent organic intermediates or polymeric byproducts on catalytic active sites [59,60], potential structural modifications due to repeated oxidative stress, and possible alterations in surface properties affecting the PMS activation efficiency [61]. This progressive decline suggests that, although Z-LDH maintains optimal catalytic performance for approximately two operational cycles, its subsequent use may require regeneration treatments to restore full activity. These findings provide important insights into the practical limitations and operational lifespan of Z-LDH catalysts for AOPs.

This leaching behavior is correlated with the gradual disintegration of the LDH structure under repetitive redox cycling, as suggested by the post-reaction XPS, which showed changes in metal oxidation states and crystallinity.

To evaluate the catalytic performance of Z-LDH, its key metrics for oxidizing and degrading BPA were compared with those of other recently reported materials in Table 1. Exceptional Reaction Kinetics: Z-LDH achieved complete degradation (100%) of BPA within 20 min, a rate that is twice as fast as its precursor, Cu_3_Mn-LDH (40 min). Outstanding Mineralization Capability: A remarkable 97% TOC removal was attained within 20 min using Z-LDH, one of the highest efficiencies among the compared materials. Efficient Catalyst Utilization and Competitive Stability: The catalyst dosage for Z-LDH (0.15 g·L^−1^) is considerably lower than that of 0.5BFO-LDHs (0.4 g·L^−1^) and FeNi-LDH@biochar (0.50 g·L^−1^), indicating a higher catalytic efficiency per mass unit. Regarding reusability, Z-LDH maintained 80% of its initial activity after three cycles, a stability on par with CoFe-LDH/S(IV) and FeNi-LDH@biochar. Although the stability is slightly lower than that of Cu_3_Mn-LDH (97% after 4 cycles) and 0.5BFO-LDHs (96.9% after 5 cycles), this is counterbalanced by Z-LDH’s dramatically enhanced reaction rate and mineralization efficiency. Crucially, unlike the CoFe-LDH system, Z-LDH avoids the use of ecotoxic cobalt, offering a more environmentally benign profile.

#### 3.3.9. Metal Escape Experiment

**Figure 14:**
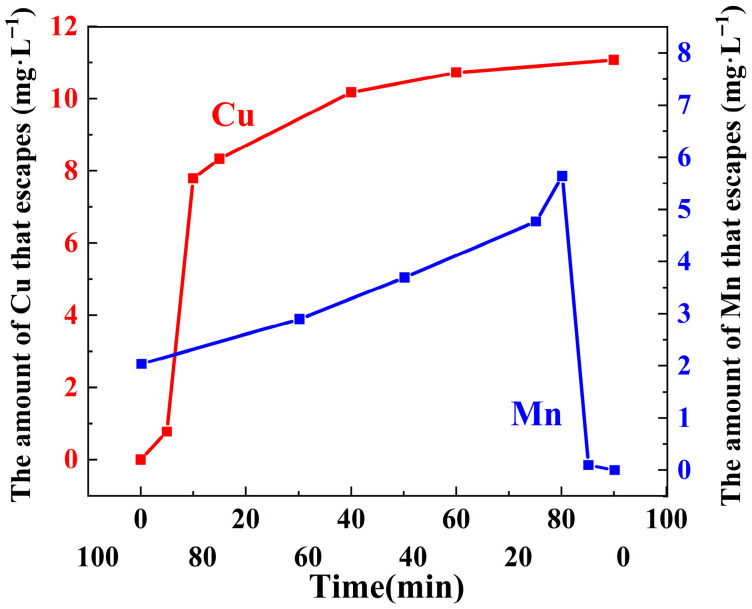
The amount of metal ions escaped during the reaction.

The metal leaching behavior of the Z-LDH composite during the reaction was evaluated. As shown in Figure 14, Cu ions were continuously released into the solution over time. Previous studies have shown that [64,65], the leaching concentrations of Cu and Mn ions generally increase under lower pH conditions. The Z-LDH composite showed a significant reduction in Cu leaching, from 36 mg·L^−1^ to 11 mg·L^−1^, demonstrating the effective role of zeolite in suppressing copper release. In contrast, the leaching behavior of Mn differed, exhibiting an initial increase followed by a decrease, with an overall higher leaching level than that of the pristine Cu_3_Mn-LDH. This suggests that Mn ions released into the solution may be re-adsorbed onto the composite or consumed through reaction with PMS. Although zeolite loading considerably reduced Cu leaching, the final concentration of Cu (11 mg·L^−1^) still exceeded the discharge limit for total copper (0.5–2.0 mg·L^−1^) set by the Chinese national standard GB 25467-2010 [66]. Similarly, the Mn concentration after reaction was also above the regulatory limit (1.0–2.0 mg·L^−1^). These results indicate that further material optimization is necessary to minimize metal leaching and meet environmental standards. A gradual increase in the leaching concentrations of both Cu and Mn ions was observed with increasing cycle numbers, as quantified by ICP-OES analysis [59]. This progressive loss of active metal species indicates the gradual disintegration of the LDH structure under repetitive reaction conditions, leading to a corresponding decline in catalytic performance. Similar phenomena of cumulative metal leaching and consequent activity loss during cycling have been widely reported in PMS-activated LDH systems [59,67].

### 3.4. Possible Catalytic Mechanism for BPA Removal

#### 3.4.1. Identification of ROS

**Figure 15:**
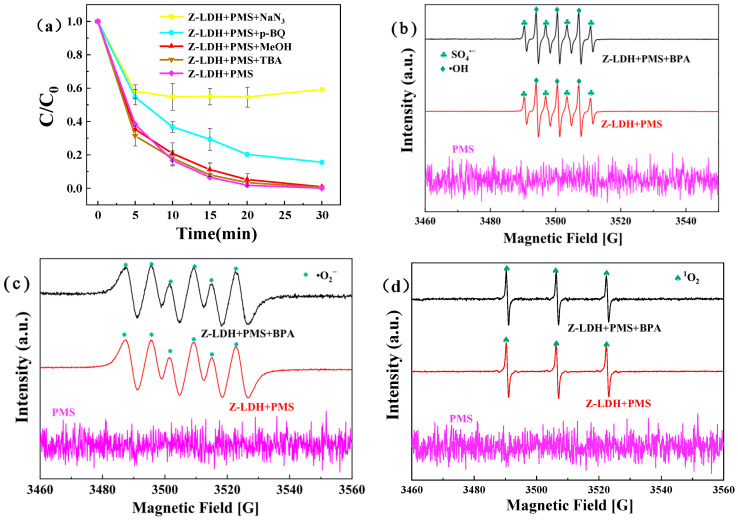
Effects of different quenchers on BPA degradation in Z-LDH/PMS system (**a**), EPR spectra of DMPO-SO_4_^•−^ and DMPO-•OH (**b**), EPR spectrum of DMPO-•O^2−^ (**c**), EPR spectrum of TEMP-^1^O_2_ (**d**), (Experimental conditions: PMS concentration = 0.15 g⋅L^−1^, Z-LDH concentration = 0.10 g⋅L^−1^, BPA concentration = 10 mg⋅L^−1^, [TEMP] =100 mM, initial pH = 7.0 ± 0.1, temperature =25 °C. [MeHO] = 250 mM⋅L^−1^, [TBA] = 250 mM⋅L^−1^, [p-BQ] = 10 mM⋅L^−1^, [NaN_3_] = 10 mM⋅L^−1^).

To elucidate the ROS involved in BPA degradation mediated by the Z-LDH+PMS system, a comprehensive quenching study was conducted using specific inhibitors. As illustrated in Figure 15a, MeOH, TBA, p-benzoquinone (p-BQ), and sodium azide (NaN_3_) were employed as selective SO_4_^•−^, •OH, •O_2_^−^, and ^1^O_2_ quenchers, respectively. The experimental results revealed distinct inhibition patterns: NaN_3_ demonstrated the most pronounced inhibitory effect, reducing the degradation efficiency to 40% after 30 min of reaction, whereas p-BQ exhibited a moderate retardation effect on the degradation rate. In contrast, both MeOH and TBA had a negligible influence on BPA degradation efficiency, suggesting minimal involvement of SO_4_^•−^ and •OH radicals in the reaction pathway. These observations collectively indicate that •O_2_^−^ and ^1^O_2_ were the predominant ROS responsible for BPA degradation in this system. These findings are in excellent agreement with our previous experimental results and relevant literature reports [21,68], confirming the coexistence of radical and non-radical degradation mechanisms in AOPs.

These observations collectively indicate that •O_2_^−^ and ^1^O_2_ were the predominant ROS responsible for BPA degradation, while the contributions of SO_4_^•−^ and •OH were minimal. It is noteworthy that the negligible quenching effects of MeOH and TBA do not necessarily imply the complete absence of SO_4_^•−^ and •OH, as confirmed by the EPR analysis (Figure 15b). Instead, this suggests that these radical species are likely short-lived intermediates that undergo rapid transformation into other reactive species (e.g., ^1^O_2_) or are consumed in side reactions before they can directly attack BPA molecules. Therefore, although SO_4_^•−^ and •OH are generated in the system, they are not the primary contributors to the degradation process. To further verify the experimental quenching results, EPR spectroscopy was used to identify the reactive ROS generated in the Z-LDH+PMS system. The EPR measurements were conducted under three distinct conditions: (1) PMS alone, (2) Z-LDH + PMS, and (3) Z-LDH + PMS in the presence of BPA. The spectra were recorded after a predetermined reaction time to evaluate the ROS contribution across the systems. As shown in Figure 15b, the characteristic signals of SO_4_^•−^ and •OH were predominantly identified detected in the Z-LDH+PMS system. Notably, these signals persisted in the Z-LDH+PMS+BPA system, suggesting that although BPA consumed ROS, it did not significantly alter the EPR signatures. This observation is consistent with the quenching experiments, wherein MeOH and TBA exhibited negligible inhibitory effects on BPA degradation. However, the detection of SO_4_^•−^ and •OH radicals via EPR implied that these species did not directly participate in BPA degradation [21].Instead, they possibly served as intermediates that underwent further transformation, ultimately facilitating ^1^O_2_ generation, which played a dominant role in the degradation [69]. Further evidence is obtained from Figure 15c,d, where distinct EPR signals for •O_2_^−^ and ^1^O_2_ were observed, serve as direct proof of their involvement in the system. However, the EPR spectrum of PMS alone exhibited no discernible radical signals, indicating that PMS did not spontaneously generate ROS capable of degrading BPA. Collectively, these findings demonstrate that Z-LDH activation is essential for PMS-mediated radical generation. Based on the results of both quenching experiments and EPR analysis, the Z-LDH+PMS system generates multiple SO_4_^•−^, •OH, •O_2_^−^, and ^1^O_2_, among which •O_2_^−^, and ^1^O_2_ play predominant roles in BPA degradation.

#### 3.4.2. XPS Analysis

**Figure 16:**
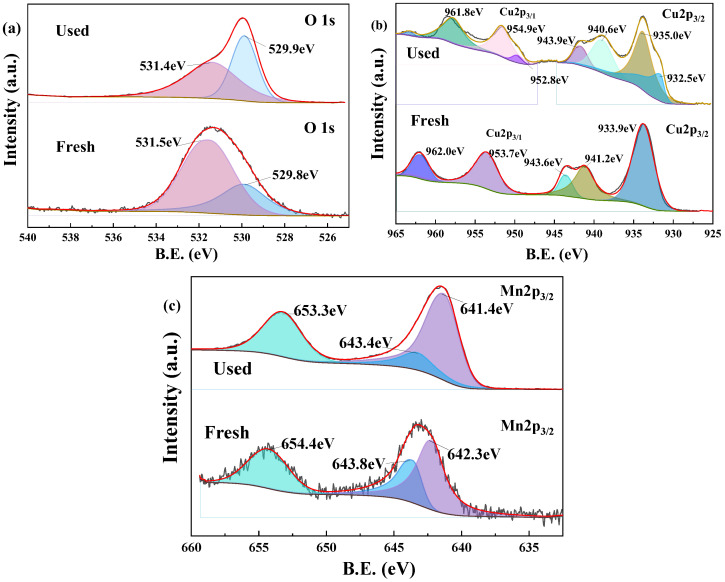
XPS spectra of Z-LDH before and after BPA degradation of O1s (**a**), Cu2p (**b**), Mn (**c**). (The open black circles represent the raw data, the red line presents the total fit, and the filled curves in various colors indicate the individual chemical components).

XPS was employed to characterize evolution of the elemental composition and oxidation states of the Z-LDH materials during PMS activation for BPA degradation. Figure 16a reveals two distinct O species: hydroxyl groups (-OH) at 531.5–531.4 eV [70], and lattice oxygen (O^2−^) at 529.8–529.9 eV [71]. Post-reaction analysis demonstrated a marked attenuation of the -OH signal intensity, suggesting either direct involvement in PMS activation or a reaction with BPA molecules. Concurrently, the enhanced lattice oxygen signal implies surface reconstruction for forming Cu-O and Mn-O bonds via the participation of metal cations.

Figure 16b shows the XPS spectra of Cu before and after the Z-LDH catalyzed PMS degradation of BPA. Cu speciation analysis exhibited characteristic Cu 2p3/2 peaks at 932.5 eV (Cu^+^), 933.9 eV (Cu^+^ in complex form) [72,73], and 935 eV (Cu^2+^), with corresponding satellite features between 940.6 and 943.9 eV. The post-catalytic spectra show a pronounced decrease in Cu^+^ (932.5 eV) concurrent with Cu^2+^ (935 eV) signal enhancement, confirming oxidative conversion of Cu^+^ → Cu^2+^ through single-electron transfer during PMS activation.

Mn redox chemistry (Figure 16c) displays characteristic Mn^3+^ (641.4–642.3 eV) [74] and Mn^4+^ (643.4–643.8 eV) [75], analogous to Cu_3_Mn-LDH systems [21]. The reaction-induced attenuation of the Mn^4+^ features with concomitant Mn^3+^ signal amplification reveals a dynamicMn^4+^/Mn^3+^ redox cycle. This valence-state interconversion facilitates continuous electron shuttling, thereby synergistically enhancing both oxidative PMS activation and reductive metal center regeneration.

#### 3.4.3. Analysis of the Degradation Pathway of BPA by Z-LDH+PMS

In the Z-LDH+PMS system for BPA degradation, multiple ROS including •OH, SO_4_^•−^, and ^1^O_2_ were involved, with ^1^O_2_ playing the dominant role and O_2_^−^ exhibiting a secondary contribution. As summarized in Equations (1)–(10), the activation of PMS by Z-LDH proceeds through a series of redox reactions and radical transformations. Initially, Cu^+^ reacted with PMS to generate Cu^2+^ and SO_4_^•−^ (Equation (1)). The resulting SO_5_^•−^ (Equation (2)) underwent spontaneous decomposition to produce ^1^O_2_ (Equation (3)). Simultaneously, SO_4_^•−^ reacted with H_2_O to form •OH (Equation (4)) [76], which can further interact with SO_4_^•−^ to yield O_2_ (Equation (5)) [77]. The generation of •O_2_^−^ is associated with electron transfer processes involving molecular oxygen (Equation (6)) [78]. Notably, •O_2_^−^ can react with •OH to form ^1^O_2_ (Equation (7)) [79], whereas the self-combination of •O_2_^−^ also contributed to ^1^O_2_ production (Equation (8)) [80]. These interconnected pathways collectively promote substantial generation of ^1^O_2_, which significantly enhances the efficiency and kinetics of BPA degradation.

XPS analysis confirmed a distinct shift in the Mn oxidation state post-reaction, suggesting that electron-rich Mn species directly participate in activating PMS for BPA degradation [81]. The catalytic process is driven by the dynamic redox cycling between Mn^4+^ and Mn^3+^, where Mn^4+^ reacts with HSO_5_^−^ to form Mn^3+^ and SO_5_^•−^ (Equation (9)), and Mn^3+^ subsequently reacts with HSO_5_^−^ to generate SO_4_^2−^ and •OH (Equation (10)) [82]. This continuous Mn^4+^/- Mn^3+^ redox cycle facilitates efficient electron transfer and, promotes the generation of diverse ROS. These ROS, engage in further radical transformation pathways, ultimately leading to the generation of ^1^O_2_, that is critical for BPA degradation of BPA. Persistent redox cycling not only sustains the catalytic activity but also improves the overall efficiency of the degradation process.

Cu^+^ + HSO_5_^−^→Cu^2+^ + SO_4_^•−^ + •OH (1)

Cu^2+^ + HSO_5_^−^→Cu^+^ + SO_5_^•−^ + H^+^ (2)

2SO_5_^•−^→2SO_4_^2−^ + ^1^O_2_ (3)

SO_4_^•−^ + H_2_O→H^+^ + SO_4_^2−^ + •OH (4)

2SO_4_^•−^+2OH^−^→2SO_4_^2−^ + 2•OH + O_2_ (5)

O_2_ + e^−^→•O_2_^−^ (6)

•O_2_^−^+•OH→OH^−^ + ^1^O_2_ (7)

•O_2_^−^ + •O_2_^−^ + 2 H^+^→^1^O_2_ + H_2_O_2_ (8)

Mn^4+^ + HSO_5_^−^→Mn^3+^ + SO_5_^•−^ + H^+^ (9)

Mn^3+^ + HSO_5_^−^→Mn^4+^ + SO_4_^2−^ + •OH (10)

**Figure 17:**
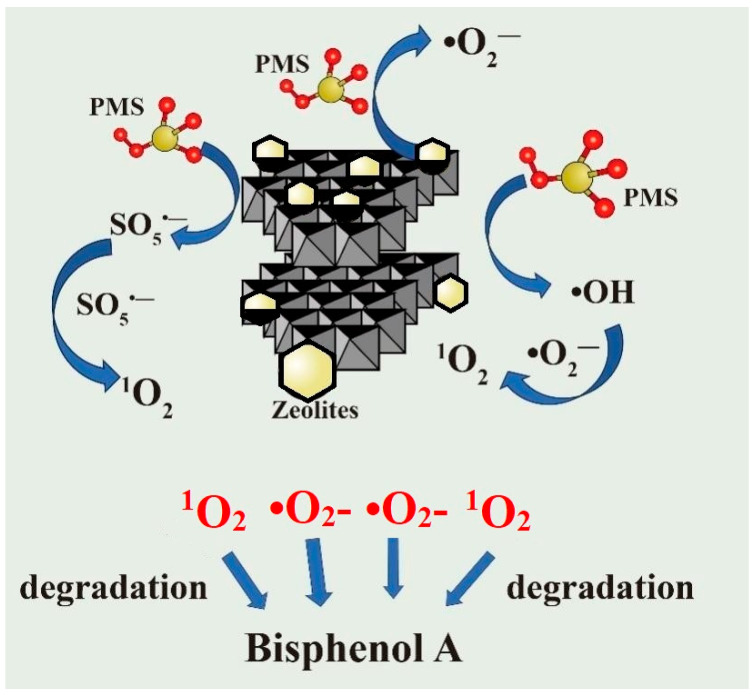
Mechanism of BPA degradation by Z-LDH+PMS. (The curved arrow depicts the activation of PMS to generate radical species, with the straight arrow denoting the subsequent degradation of BPA by these radicals.).

**Figure 18:**
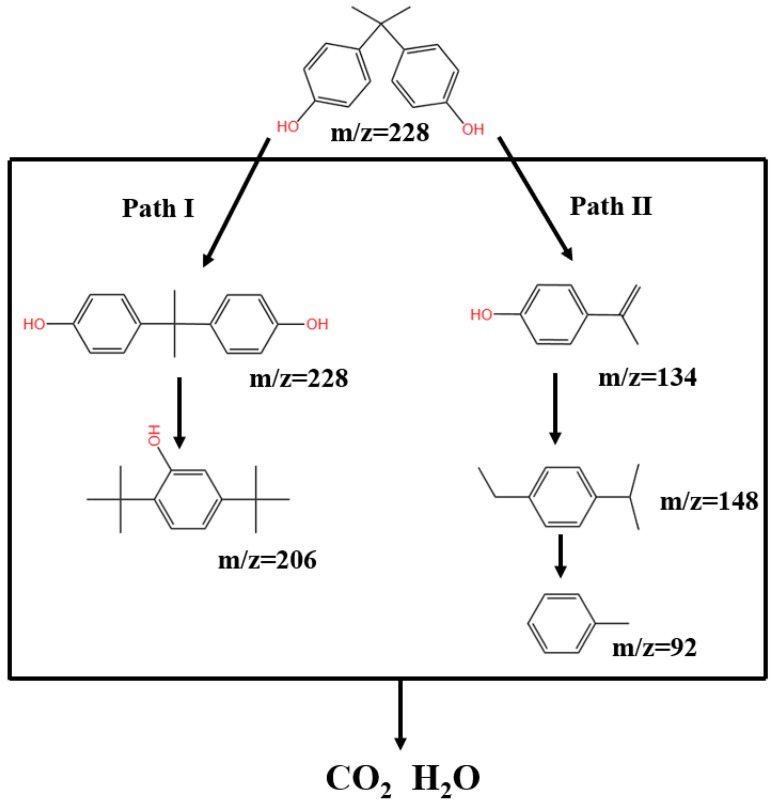
The pathways of BPA degradation induced in the Z-LDH+PMS system.

The BPA degradation mechanism in the Z-LDH/PMS system is illustrated in Figure 17. Mineralization studies (Figure 12) demonstrate that Z-LDH exhibits excellent mineralization capability for BPA, although intermediate products remain detectable during the process. GC-MS analysis was conducted at 5 and 20 min intervals (Figure S4) to identify the degradation products and elucidate the oxidative degradation pathway. Five key intermediates were identified by chromatographic analysis: 2,5-di-tert-butylphenol (*m*/*z* = 206), 1-ethyl-4-isopropylbenzene (*m*/*z* =148), 4-isopropylphenol (*m*/*z* = 134), and toluene (*m*/*z* = 92). Based on these intermediates and the existing literature, we propose two primary degradation pathways (Figure 18). In Pathway I, ROS attack BPA, resulting in Carbon chain cleavage and the formation of 2,5-di-tert-butylphenol. Pathway II involves progressive oxidation and degradation of the C skeleton, followed by dihydroxylation, to ultimately yield toluene (*m*/*z* = 94). The oxidative degradation process is characterized by a successive reduction in the molecular weight of the intermediate products, culminating in complete mineralization to H_2_O and CO_2_ [83,84]

## 4. Conclusions

In this study, a high-performance Z-LDH composite catalyst was successfully developed by uniformly dispersing Cu_3_Mn-LDH onto the zeolite surfaces, demonstrating the exceptional efficiency of PMS activation for BPA removal. The Z-LDH+PMS system achieved ultrafast degradation of 10 mg⋅L^−1^ BPA within 20 min under optimal conditions (0.15 g⋅L^−1^ catalyst with 0.15 g⋅L^−1^ PMS), accompanied by a remarkable 97.0% TOC removal efficiency. The composite exhibited outstanding degradation performance and robust stability under diverse environmental conditions, including varying temperatures, coexisting anions, wide pH ranges, and varying pollutant concentrations. The superior catalytic activity originated from the synergistic Cu^+^/Cu^2+^ and Mn^4+^/Mn^3+^ redox cycles in the LDH structure, thereby promoting generation of ROS, with •O_2_^−^ and ^1^O_2_ as the dominant contributors. The incorporation of zeolite significantly enhanced the electron transfer efficiency, thereby accelerating the BPA degradation kinetics. GC-MS analysis identified five key degradation intermediates, suggesting two plausible pathways for completely decomposing BPA to CO_2_ and H_2_O. This study developed a high-performance LDH-based catalysts and elucidated its degradation mechanism, with significant potential for practical applications in the remediation of emerging contaminants.

## Figures and Tables

**Table 1:** Comparison of properties of different materials.

Material	Degradation Efficiency	TOC Removal Rate	Dosage	Loop Count	Source
Z-LDH	100% within 20 min	97% within 20 min	[Z-LDH] = 0.15 g·L^−1^, [PMS] = 0.25 g·L^−1^, [BPA] = 10 mg·L^−1^	80% after 3 cycles	This work
Cu_3_Mn-LDH	100% within 40 min	95% within 40 min	[Cu_3_Mn-LDH] = 0.10 g·L^−1^, [PMS] = 0.25 g·L^−1^, [BPA] = 10 mg·L^−1^	97% after 4 cycles	[21]
0.5BFO-LDHs	99.3% within 15 min (with light)	97.6% within 15 min	[BFO-LDHs] = 0.4g·L^−1^, [PMS] = 0.4 mmol·L^−1^, [BPA]_0_ = 20 mg·L^−1^	96.9% after 5 cycles	[62]
CoFe-LDH/S(IV)	84.8% within 20 min	Not reported	[CoFe-LDH] = 0.1 g·L^−1^, [S(IV)] = 1.5 mmol·L^−1^, [BPA]_0_ = 2 μmol·L^−1^	80% after 5 cycles	[63]
FeNi-LDH@biochar	Not specified (60% TOC removal in 120 min)	60% within 120 min	[FeNi-LDH@biochar] = 0.50 g·L^−1^, [PMS] = 0.75 g·L^−1^, [DOX]_0_ = 35 mg·L^−1^	80% after 3 cycles	[57]

## Data Availability

The data are available in the article and its Supplementary Materials.

## Supplementary Materials

The following supporting information can be downloaded at: https://www.mdpi.com/article/10.3390/toxics13100889/s1. Figure S1: EDS analysis of Z-LDH; Figure S2: Z-LDH Transmission Electron Microscopy; Figure S3: Z-LDH Transmission Electron Microscopy; Figure S4: The GC/MS chromatogram of BPA degradation intermediates in the Z-LDH; Table S1: The specific surface area and aperture ratio of the material.
