# Supplementary material for: Advanced Catalytic Peroxymonosulfate Activation via Zeolite-Supported Cu3Mn-Layered Double Hydroxide for Enhanced Oxidative Degradation of Bisphenol A (BPA)
Source: Toxics. 2025 Oct 17;13(10):889. doi: 10.3390/toxics13100889 (PMC12568224; doi:10.3390/toxics13100889)
Supplement: Supplementary file 1 [file toxics-13-00889-s001.zip › toxics-3879025-supplementary.pdf]

Advanced Catalytic Peroxymonosulfate Activation via Zeolite-Supported Cu_3Mn-Layered Double Hydroxide for Enhanced Oxidative Degradation of Bisphenol A (BPA)

Qiuyi Li [1,2], Chongmin Liu [1,2,3,4,*], Meina Liang [1,2,3,4,*], Mi Feng [1,2,3,4], Zejing Xu [1,2], Dunqiu Wang [1,2,3,4] and Saeed Rad [1,2,3]

1 College of Environmental Science and Engineering, Guilin University of Technology, Guilin 541004, China; lqy1626743718@163.com (Q.L.)

2 Guangxi Key Laboratory of Theory & Technology for Environmental Pollution Control, Guilin University of Technology, Guilin 541004, China

3 University Engineering Research Center of Watershed Protection and Green Development, Guangxi, Guilin University of Technology, Guilin 541006, China

4 Key Laboratory of Carbon Emission and Pollutant Collaborative Control, Education Department of Guangxi Zhuang Autonomous Region, Guilin University of Technology, Guilin 541006, China

* Corresponding author: chongmin@glut.edu.cn (C.L.); liangmeinaa@163.com (M.L.)

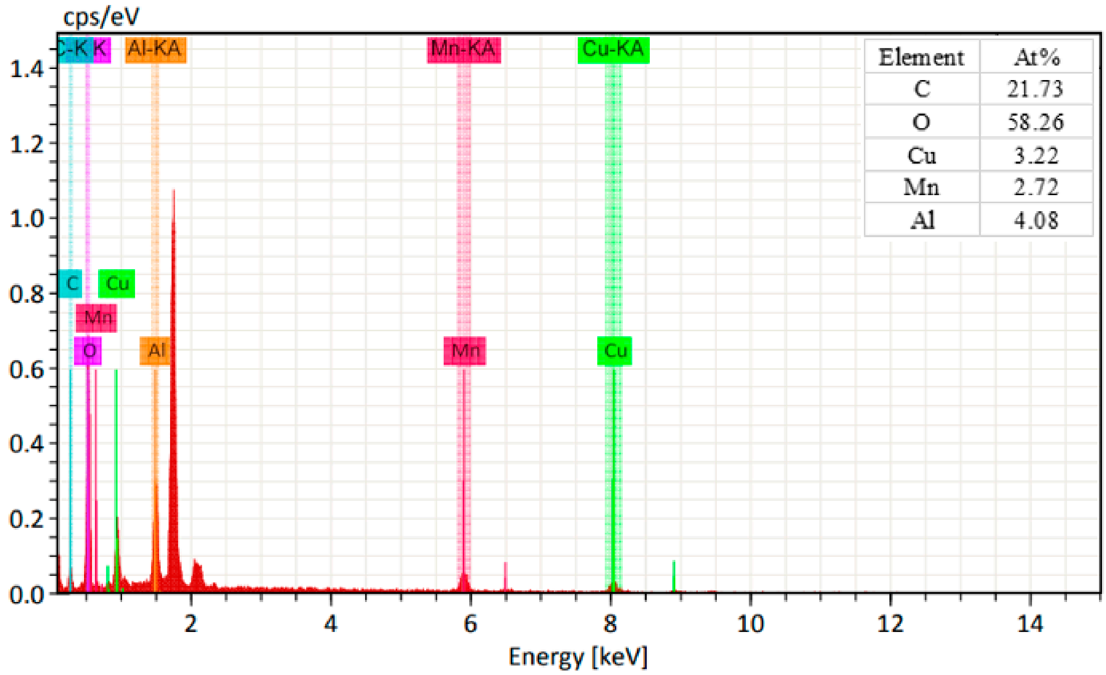

Figure S1. EDS analysis of Z-LDH.

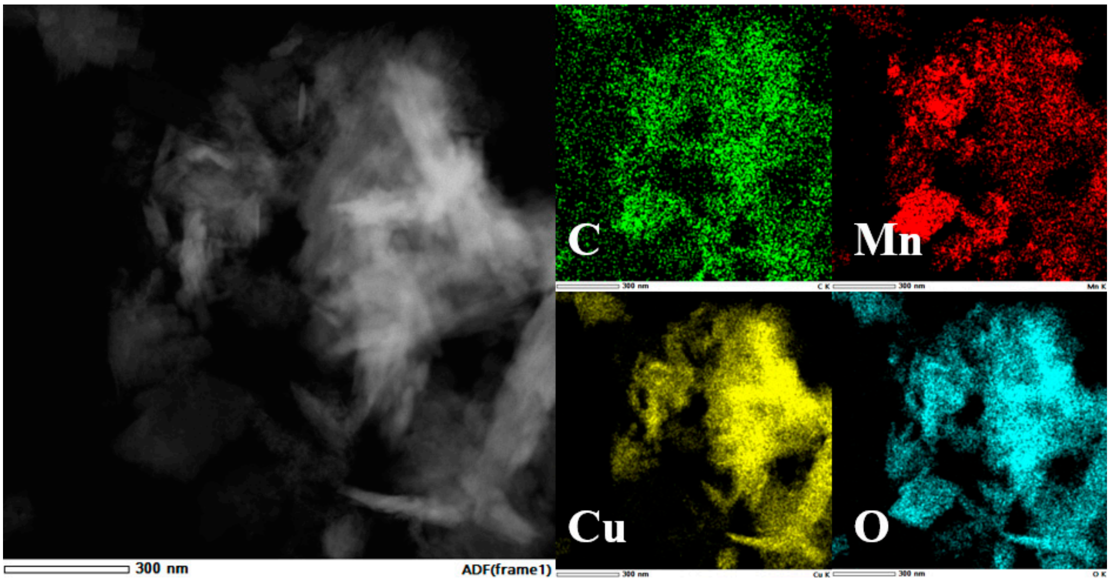

Figure S2. Z-LDH Transmission Electron Microscopy.

Table S1. The specific surface area and aperture ratio of the material.

Materials	$S_{BET}/(m^2/g)$	$V_{total}/(cm^3/g)$	D_{BET}/nm
Zeolite	36.46	0.079	17.90
Z-LDH	122.72	0.231	12.36

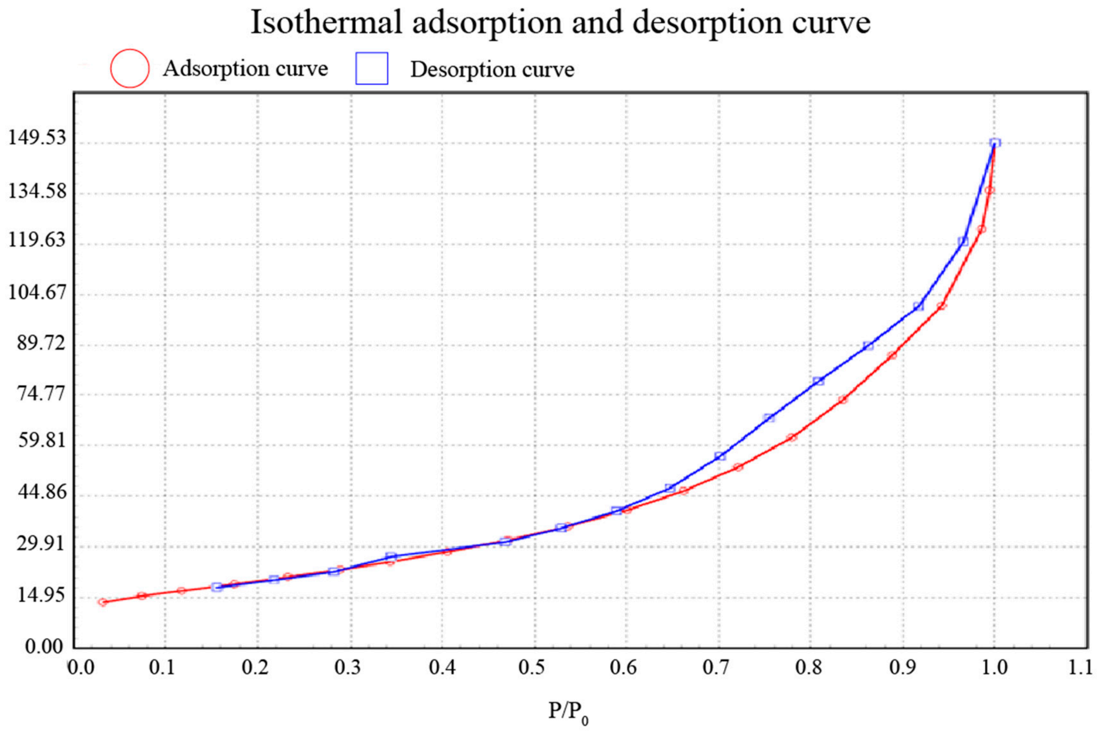

Figure S3. Z-LDH Transmission Electron Microscopy.

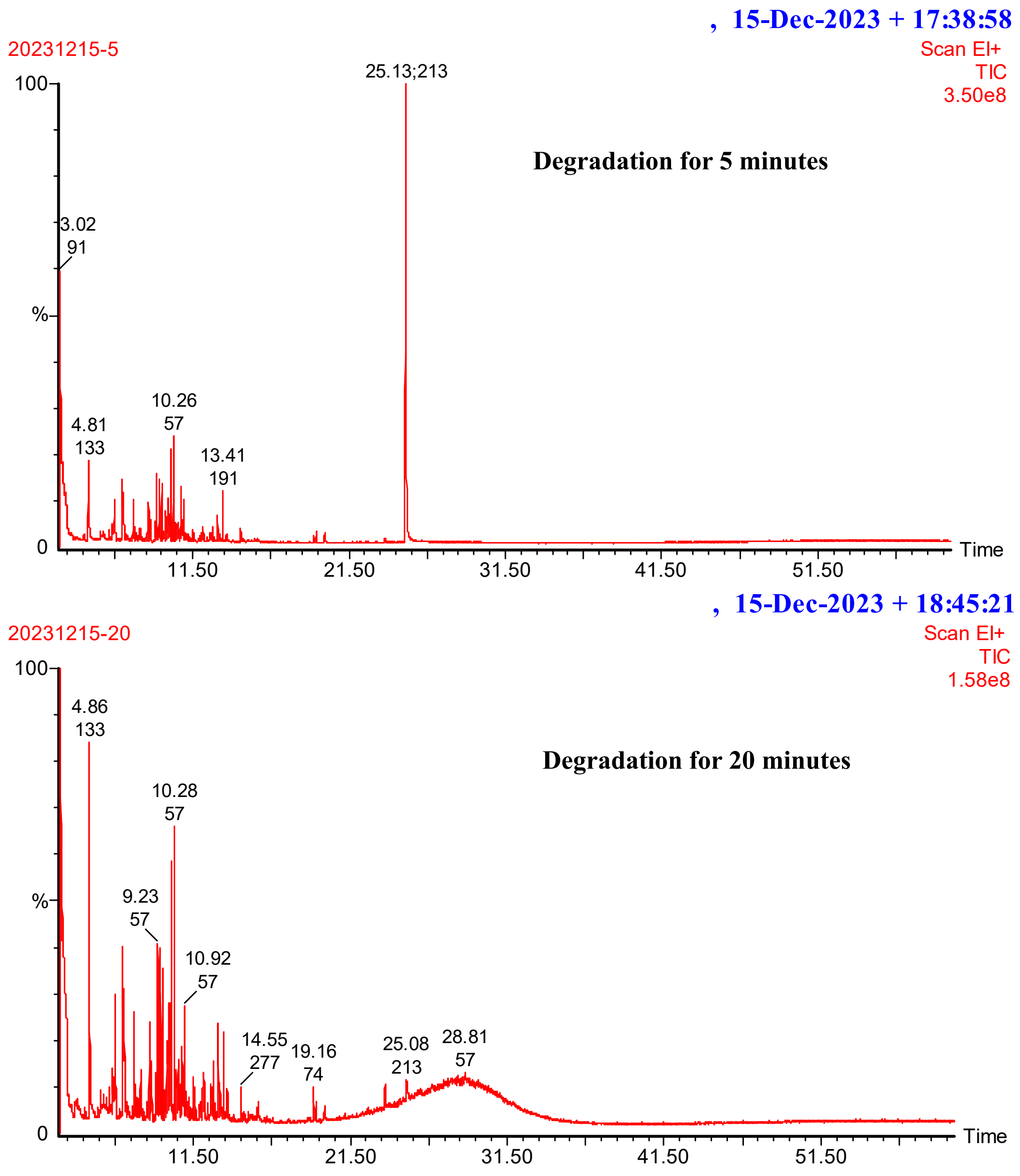

Figure S4. The GC/MS chromatogram of BPA degradation intermediates in the Z-LDH.
